# Research Progress on Biomimetic Water Collection Materials

**DOI:** 10.3390/biomimetics11010067

**Published:** 2026-01-13

**Authors:** Hengyu Pan, Lingmei Zhu, Huijie Wei, Tiance Zhang, Boyang Tian, Jianhua Wang, Yongping Hou, Yongmei Zheng

**Affiliations:** Key Laboratory of Bio-Inspired Smart Interfacial Science and Technology of Ministry of Education, School of Chemistry, Beihang University (BUAA), Beijing 100191, China

**Keywords:** biomimetic water collection materials, Laplace pressure difference, directional droplet transport, heterogeneous wettability

## Abstract

Water scarcity constitutes a major global challenge. Biomimetic water collection materials, which mimic the efficient water capture and transport mechanisms, offer a crucial approach to addressing the water crisis. This review summarizes the research progress on biomimetic water collection materials, focusing on biological prototypes, operational mechanisms, and core aspects of biomimetic design. Typical water-collecting biological surfaces in nature exhibit distinctive structure–function synergy: spider silk achieves directional droplet transport via periodic spindle-knot structures, utilizing Laplace pressure difference and surface energy gradient; the desert beetle’s back features hydrophilic microstructures and a hydrophobic waxy coating, forming a fog-water collection system based on heterogeneous wettability; cactus spines enhance droplet transport efficiency through the synergy of gradient grooves and barbs; and shorebird beaks enable rapid water convergence via liquid bridge effects. These biological prototypes provide vital inspiration for the design of biomimetic water collection materials. Drawing on biological mechanisms, researchers have developed diverse biomimetic water collection materials. This review offers a theoretical reference for their structural design and performance enhancement, highlighting bio-inspiration’s core value in high-efficiency water collection material development. Additionally, this paper discusses challenges and opportunities of these materials, providing insights for advancing the engineering application of next-generation high-efficiency biomimetic water collection materials.

## 1. Introduction

The uneven global distribution of water resources coupled with the intensification of climate change has made the problem of water scarcity in arid and semi-arid regions increasingly severe [1,2,3,4,5]. Traditional water collection technologies, such as seawater desalination, fog nets, and reservoirs [6,7,8], are constrained by bottlenecks including limited fog droplet capture efficiency, poor adaptability to complex environments (e.g., high temperatures, strong sandstorms), and high costs for large-scale fabrication [6,7,9,10,11]. These limitations make it difficult to meet the demand for efficient and stable water collection in practical applications, thus driving the development of novel water collection materials as a key research direction to overcome this predicament [12].

**Figure 1:**
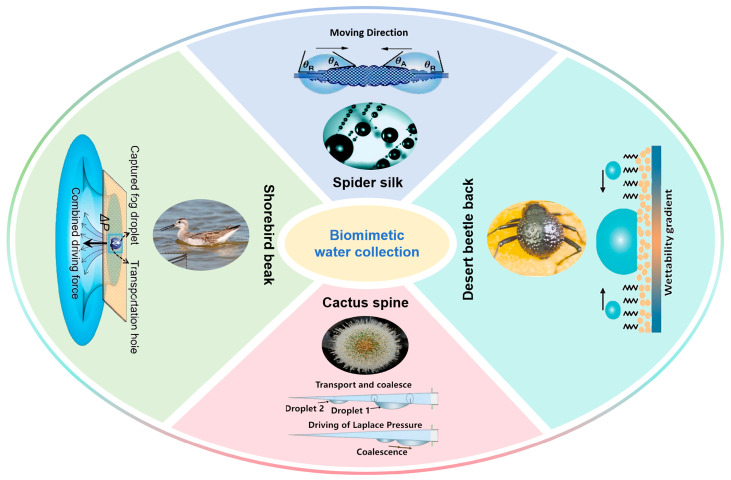
Biological surfaces with water collection function: spider silk, shorebird beak, cactus spine, and desert beetle back [30,31,32,33,34].

Over long-term adaptation to extreme water-scarce environments, various organisms in nature have evolved unique surface structures and wetting regulation mechanisms for efficient water collection, serving as natural and high-performance biological templates for the design of biomimetic water collection materials [13,14,15,16,17,18,19,20,21,22,23,24,25,26,27,28,29]. As shown in Figure 1, typical organisms include spider silk, the backs of desert beetles, cactus spines, and shorebird beaks [30,31,32,33,34]. Spider silk features a periodic “spindle-knot–joint” structure, enabling efficient fog droplet capture. The difference in roughness between spindle knots and joints generates geometric gradients and surface energy gradients, which synergistically interact with Laplace pressure differences to drive the convergence of liquid droplets toward the spindle knots [35,36,37,38,39,40,41,42]. The elytra of desert beetles are covered with “mountain-peak-shaped” microprotrusions: the tops of these protrusions are wax-free superhydrophilic regions that rapidly adsorb tiny fog droplets, while the “valley” regions between protrusions are superhydrophobic due to waxy coatings, guiding aggregated droplets to flow directionally into the beetles’ mouths [30,43,44,45,46,47,48,49,50,51]. Cactus spines exhibit a three-segment structure consisting of tip barbs, middle gradient grooves, and base belt-like trichomes. The barbs capture fog droplets, the middle gradient grooves guide droplets toward the base via capillary force and Laplace pressure difference, and the base trichomes enable temporary water storage [34,52,53,54,55,56]. Shorebirds form a V-shaped wedge structure with their upper and lower jaws. During periodic opening and closing of the beak, liquid bridge effects and interfacial tension regulation, combined with Laplace pressure difference induced by the beak’s geometric structure, shorebirds achieve the rapid and directional transport of fog droplets to the oral cavity [33,57,58]. The water collection strategies of these organisms all reflect a high degree of “structure–function” synergy, providing critical inspiration for the development of biomimetic water collection materials [59].

However, current research still faces numerous challenges. Achieving accurate replication and large-scale fabrication of biomimetic structures is challenging, the stability of materials in complex natural environments needs to be improved, and a standardized efficiency evaluation system has not yet been fully established. The development of reliable and standardized measurement systems is crucial for the accurate evaluation and comparison of the performance of different fog water collection materials. Recent studies have reported the development of a dedicated device for evaluating the fog harvesting efficiency of fibrous collectors, enabling real-time measurement [60]. Meanwhile, researchers have conducted extensive explorations in the regulation of material wettability and structural optimization. For instance, multilayer harp structures can maximize the contact probability between fog flow and the collection surface by optimizing interlayer spacing and fiber arrangement, providing new insights for the design and development of future materials [61].

Centering on the typical biological water collection systems illustrated in Figure 1, this review systematically summarizes the water collection structures, wetting properties, and core mechanisms of spider silk, desert beetle backs, cactus spines, and shorebird beaks. It further analyzes the design principles of biomimetic water collection materials, discusses the processes of fog droplet capture and directional transport in these materials, and reviews their application progress. Additionally, the review explores the challenges faced by current biomimetic water collection materials in terms of efficiency, stability, and large-scale application, aiming to promote the translation of biomimetic water collection materials from laboratory research to practical use and provide technical support for addressing global water scarcity.

## 2. Water Collection Mechanisms of Biomimetic Materials

**Figure 2:**
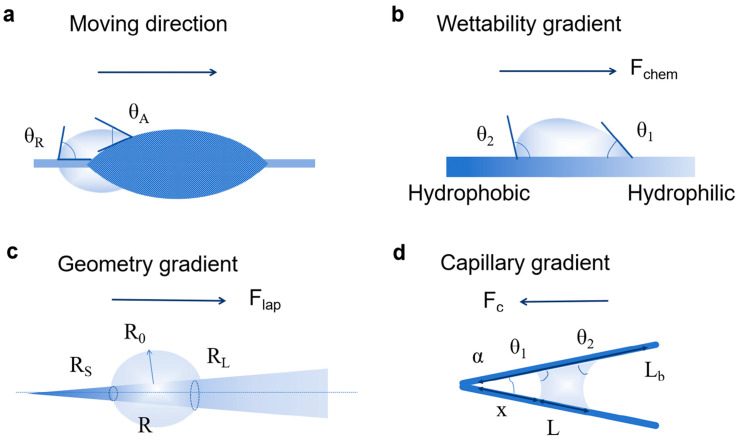
Illustration of the directional water collection mechanisms of biomimetic materials: (**a**) Mechanism of directional water collection in wetted spider silk; (**b**) Driving force for directional liquid transportation induced by wettability gradient; (**c**) Geometric gradient-induced driving forces for directional fluid transport; (**d**) Capillary gradient for directional water collection in shorebird beaks.

The water collection process encompasses four critical stages: nucleation, consolidation, transport, and storage. The capture and directional motion of water droplets are intrinsically governed by surface wettability, which is predominantly determined by both surface chemical composition and topographic roughness. Numerous theoretical models, derived from biological prototypes, have been developed to elucidate the mechanisms of fog collection in nature, as illustrated in Figure 2. These models provide fundamental insights into the fog harvesting process, essentially explaining the static and dynamic interfacial contact behaviors of droplets.

Figure 2a illustrates the directional water collection mechanism of wet spider silk. The spindle-knots of the silk have a random nanofiber structure, while the joints have an aligned nanofiber structure. The former exhibits higher roughness, stronger surface hydrophilicity, and higher surface energy. According to Wenzel’s law:

(1) $$\cos\;\theta_{w\;}=\;r\;\cos\;\theta$$

where *r* is surface roughness, *θ_w_* and *θ* are the apparent and intrinsic contact angles on rough and smooth surfaces. The roughness difference between the two forms a geometric gradient and a surface energy gradient, generating a driving force toward the spindle-knots. The force generated by a surface energy gradient that arises from a difference in surface roughness is given by:

(2) $$F_{s\;}=\;\int_{L_{j}}^{L_{s}}\gamma \;\left(\cos\theta_{A}\text{-}\cos\theta_{R}\right)\mathrm{dl}$$

where *γ* is the surface tension of water, *θ_A_* and *θ_R_* are the advancing and receding angles of water drop on spider silk, respectively. And *dl* is the integrating variable along the length from the joint (*L_j_*) to the spindle-knot (*L_s_*). Meanwhile, the radius of curvature at the joints (2*r*_1_) is smaller than that at the spindle-knots (2*r*_2_). According to the Laplace equation, the droplet pressure at the joints is higher, creating a Laplace pressure difference directed toward the spindle-knots. The driving force can be expressed as follows:

(3) $$F_{\mathrm{lap}\;}=-\int_{r_{1}}^{r_{2}}\frac{2\gamma }{{\left(r+R_{0}\right)}^{2}}\;\sin\;\varphi \;\mathrm{dR}$$

where *dR* is the infinitesimal radius increment along the spindle-knot, *r* is the local radius, *R*_0_ represents the droplet height, *r*_1_ and *r*_2_ are the local radii of the droplet on the smallest and largest sides, *φ* is the half apex-angle of the spindle-knot, respectively. Under the synergistic effect of these two driving forces $F_{s}$ and $F_{\mathrm{lap}}$, droplets on the joint surface overcome contact hysteresis resistance and move directionally toward the spindle-knots along the silk’s axial direction. During this process, the random nanofibers in the spindle-knots form a discontinuous three-phase contact line (TCL), enhancing droplet adhesion to prevent backflow. In contrast, the aligned nanofibers in the joints form a continuous TCL, reducing droplet migration resistance. Together, these features ensure stable one-way droplet transport, ultimately leading to droplet aggregation at the spindle-knots and achieving directional collection.

Figure 2b illustrates the synergistic water collection effect of different wettable regions on desert beetle backs. The driving force generated by the wettability gradient (*F_chem_*) can be characterized as follows:

(4) $$F_{chem}=\;\pi \;R_{0}\;\gamma \;\left(\cos\theta_{2}\text{-}\cos\theta_{1}\right)$$

where *R*_0_ represents the radius of the droplet. Moreover, *θ*_2_ and *θ*_1_ denote the contact angles of the droplet on the hydrophobic and hydrophilic sides, respectively. This serves as the core design prototype for biomimetic wettability-based water collection materials. The droplets on the backs of the desert beetle is sufficiently massive to roll into the wind. When the wind speed is excessively high, the rolling water droplets may likewise be dispersed into the air. When the droplet slides along the hydrophobic surface with an inclination angle of *θ*, the maximum wind speed (*V_m_*) can be calculated by the following formula:

(5) $$V_{m}\;\text{≈}\sqrt{\left[\left(\frac{\rho_{w}}{\rho_{a}}\right)\left(\frac{4}{3}\right)R\;g\;\sin\;\theta \right]}\;$$

where *R* is the radius of the droplet, *g* is the acceleration due to gravity, ρ*_w_* and ρ*_a_* are the density of the medium water and air, respectively. The biomimetic design of this fog collection structure can be commercialized and produced on a large scale at low cost, and is expected to be applied in water collection tents, building claddings, condensers, engines, and other fields.

For substrates with asymmetric geometries, such as cactus spines (Figure 2c), an unbalanced Laplace pressure will be generated to promote the directional movement of droplets. The driving force on a conical object can be expressed as follows:

(6) $$F_{Lap}=\int_{R_{S}}^{R_{L}}\frac{2\gamma }{{\left(R\;+\;R_{0}\right)}^{2}}\;\sin\varphi \;dR\;$$

where *dR* is the infinitesimal radius increment along the cone. *R* indicates the local radius of the object at any position. *R*_0_ represents the droplet height. *R_S_* and *R_L_* are the local radii of the droplet on the smallest and largest sides of the object, respectively.

For the shorebird beak, this directional transport is primarily driven by the beak’s wedge-shaped structure—the curvature difference between its leading and trailing edges generates a Laplace pressure difference on the droplet surface (Figure 2d). The contact angle of the droplet is *h*, the width of the droplet is *W*, the length of the droplet is *L*, the opening angle is *θ*, and the distance from the droplet to the base of the shorebird’s beak is X. Subsequently, the capillary force *F_c_* can be expressed as:

(7) $$F_{c}\;=\;\gamma \;\cos\;\theta \left(\frac{\mathrm{WL}}{X}\right)$$

Additionally, during the beak-opening process, the contact angle hysteresis effect forms a locking force to prevent droplet backflow.

These natural models reveal that the entire process of droplet capture, coalescence, directional transport, and final collection is governed by the wettability of the material’s surface, which is itself determined collectively by the surface’s chemical composition and microscopic roughness. A deep understanding of these theoretical models provides a solid foundation and innovative design principles for developing and optimizing high-performance biomimetic water harvesting materials.

## 3. Biomimetic Materials for Water Collection

In nature, spider silk stands as a paradigmatic biological model for efficient fog collection in water-scarce environments, with its ability to directionally capture, transport, and aggregate droplets providing critical inspiration and a scientific foundation for the structural and functional design of bioinspired water-harvesting materials. Beyond spider silk, organisms such as desert beetles, cacti, and shorebirds—each relying on unique “structure–function” synergy mechanisms embodied in their back surfaces, spines, and beaks—offer a diverse array of biological prototypes for the development of multifunctional biomimetic materials. By extracting and refining the core water-harvesting principles of these natural systems, researchers have replicated, optimized, and innovated upon their structural designs, leading to a growing suite of bioinspired materials tailored to different environmental conditions. This expanding toolkit not only deepens our understanding of interfacial fluid transport but also broadens the potential applications of bioinspired water collection technologies across real-world scenarios.

### 3.1. Biomimetic Materials Inspired by Spider Silks for Water Collection

#### 3.1.1. Biological Structure of Spider Silk

**Figure 3:**
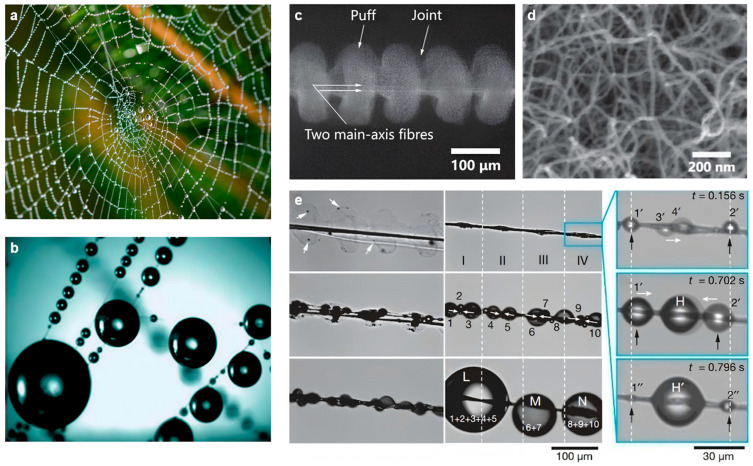
Structure of spider silk fibers and research on their water collection process: (**a**) Optical image of a naturally wetted spider web. Photo by George Rosema, Unsplash, https://unsplash.com/; (**b**) Microscopic view of droplets on a wetted spider web; (**c**) Scanning electron microscope (SEM) micrograph of spider silk; (**d**) SEM image of the puff structure, which consists of nanofibrils; (**e**) In situ microscopic visualization of the continuous water collection process of spider silk in a foggy setting —tiny droplets emerge at the spindle-knots (marked by arrows), and condensed droplets swiftly migrate and merge for collection [31].

Figure 3a presents an optical image of natural wet spider silk, with transparent water droplets uniformly attached to its surface, intuitively demonstrating its fog collection capability [39,62,63,64]. Microscopic observation of wet spider silk reveals a periodic distribution of water droplets (Figure 3b), indicating regular structural heterogeneity [65,66,67,68,69,70]. Environmental scanning electron microscopy (SEM) images of dry spider silk (Figure 3c,d) unveil its intricate structure: dry spider silk is supported by two main axial fibers, with periodically distributed puff-joint units along the axial direction. The puff structure has a diameter of approximately 130.8 μm, while the joint diameter is significantly smaller, at around 41.6 μm [71]. The magnified SEM image in Figure 3d shows that the interior of the puff structure consists of randomly interwoven nanofibers (20–30 nm in diameter), forming a three-dimensional network with a high specific surface area. This nanoscale structure enhances the contact area with fog droplets and strengthens droplet adsorption, serving as a key structural foundation for initial fog droplet capture [72]. When dry spider silk is exposed to a foggy environment, significant wet reconstruction occurs: the fluff on the silk absorbs tiny water droplets and then contracts to form a periodic spindle-knot structure. The spindle-knots are shuttle-shaped with internally random nanofibers, while the joints comprise axially aligned nanofibers. Along the silk axis, the spindle-knots exhibit greater surface roughness than the joints. This roughness difference forms a geometric gradient and a surface energy gradient, driving water droplets to move directionally from the joints to the spindle-knots, thereby achieving directional water collection [73].

Figure 3e displays in situ microscopic images of the continuous water collection process of spider silk in a foggy environment. Before exposure to fog, the spider silk is dry. The transparent fluffy structure composed of interwoven nanofibers on its surface is clearly observable under an optical microscope, and the silk exhibits no directional water collection capability at this stage. When the silk comes into contact with fog, tiny fog droplets first condense on the surface of the nanofibers in the fluffy structure. With continuous fog droplet adsorption, the initially loose fluffy structure gradually contracts and reconstructs, eventually forming periodically arranged spindle-knots and joints along the silk’s axial direction, which enables directional droplet transport. Newly generated tiny fog droplets attach randomly to the joints and spindle-knots. As the droplets continue to condense and grow, they move directionally toward the nearest spindle-knot, rapidly fuse with other migrating droplets, and gradually aggregate into large droplets. When the gravity of the large droplets exceeds the combined force of the silk’s surface tension and adhesion, the droplets fall along the silk, completing a water collection cycle. The surfaces of the joints and spindle-knots are then re-exposed, initiating the next cycle of fog droplet condensation and transport. The water collection process on a single spindle-knot is shown on the right side of Figure 3e.

#### 3.1.2. Fabrication Methods of Biomimetic Spider Silk Fiber

**Figure 4:**
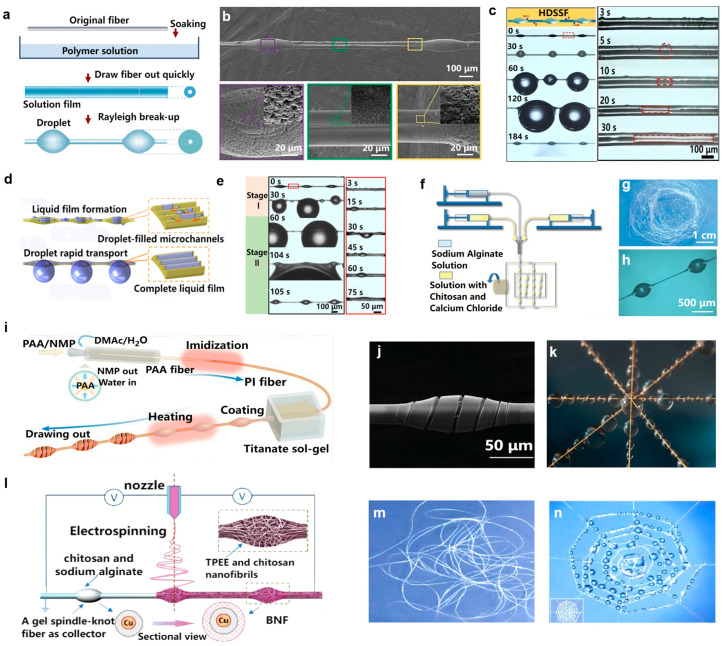
Preparation and research of biomimetic spider silk fiber materials: (**a**) Schematic representation of fabricating biomimetic spider silk fibers via the dip coating methods [63]; (**b**) SEM image of the HDSSF with a roughness gradient; (**c**) Fog collection process of HDSSF [78]; (**d**) Water collection mechanism of HMSSF; (**e**) Fog collection process of HMSSF [82]; (**f**) Schematic diagram of producing ultrafine fibers using the microfluidic approach; (**g**,**h**) Optical views of the fabricated biomimetic spider silk fibers [83]; (**i**) Schematic outline of preparing the HSK; (**j**) SEM micrograph of the HSK microfiber; (**k**) Microscopic visualization of the HSK microfiber during high-efficiency water collection [88]; (**l**) Schematic diagram of BNF preparation by electrospinning; (**m**) Optical image of the prepared BNFs; (**n**) Large-scale water collection phenomenon on BNF materials [68].

Based on the directional water collection mechanism of natural spider silk, researchers have designed and fabricated various biomimetic water-collecting spider silk materials by replicating the core structural characteristics and operational principles of its spindle-knots and joints. Currently, the most commonly used method for preparing biomimetic water-collecting spider silk fibers is the dip-coating method. Figure 4a shows a schematic diagram of the dip-coating process for preparing biomimetic spider silk fibers [63]. The original fiber is immersed in a specific polymer solution for a certain duration and then pulled out rapidly at a controlled rate, forming a uniform and viscous solution film on the fiber surface. Owing to the Rayleigh instability effect, the solution film on the fiber surface breaks into periodically distributed droplets along the axial direction. After curing, a spindle-knot–joint structure highly similar to that of natural spider silk is finally constructed on the fiber surface [74,75,76,77]. The dip-coating method offers advantages such as simple operation, controllable equipment costs, and preliminary large-scale fabrication potential, providing a process reference and experimental platform for the subsequent development of functionalized biomimetic fibers.

Researchers have prepared various biomimetic spider silk fibers for efficient water collection using the dip-coating method. Inspired by spider silk and Sarracenia trichomes [76,77,78,79,80], Huan et al. introduced capillary force and designed a novel biomimetic hydrophilic double-silk spider silk fiber (HDSSF) via dip-coating [78]. Figure 4b presents an SEM image of the HDSSF. The spindle-knot surface exhibits a rough structure with a roughness gradient from the center to the end of the knot, similar to natural spider silk. The nylon fiber between the spindle-knots remains relatively smooth and gradually becomes rough as it approaches the spindle-knots, showing anisotropic distribution due to structural extension. Similarly, HDSSF exhibits a distinct fog collection mode (Figure 4c). Tiny droplets captured on the fiber surface are drawn into the microchannels between the double-stranded fibers to form a liquid film under the influence of wettability differences. The liquid film then expands to both sides under capillary force until the microchannels are filled with water and connected to adjacent spindle-knots. Owing to the connectivity of the liquid film, when newly captured droplets come into contact with the liquid film, they rapidly merge into the large droplets on the spindle-knots under the combined action of capillary force and internal Laplace pressure difference, keeping the fiber surface free of droplets and enabling rapid droplet transport and directional aggregation.

Combining the surface wrinkling principle of highly deformable soft elastomers, Wang et al. developed a special spider silk and Sarracenia trichome shape like bioinspired fiber with hydrophilic microchannels (HMSSF) through an innovative dip-coating and mechanical stretching process [81,82]. Figure 4d illustrates the water collection mechanism of HMSSF. After a liquid film is formed in the microchannels, captured droplets can quickly converge into numerous microchannels under the drive of Laplace pressure difference and capillary force, and are transported to the center of the spindle-knots, significantly improving the transport efficiency of captured droplets. Figure 4e shows the fog collection process on the HMSSF surface, which is divided into two stages. In the first stage, captured droplets are easily drawn into the microchannels, so only a small number of droplets are observable. And in the second stage, no obvious droplets appear because the captured droplets are rapidly transported through the liquid film in the microchannels. While retaining the efficient fog capture capability of natural spider silk, HMSSF further accelerates droplet transport, achieving a synergistic improvement in fog collection efficiency.

To realize large-scale fabrication of biomimetic spider silk fibers, Liu et al. prepared heterogeneous-structured rough spindle knot microfibers (HRSFs) using a parallel nozzle microfluidic method (Figure 4f–h) [83,84]. During spinning, sodium alginate solution and chitosan solution meet at the needle tip. When the sodium alginate solution encounters calcium ions in the chitosan solution, calcium alginate gel fibers are rapidly formed. After the chitosan solution coats the sodium alginate solution [85,86,87], it breaks into small droplets due to Rayleigh instability, eventually forming a beaded structure. These fibers exhibit excellent mechanical properties and corrosion resistance, enabling large-scale preparation and efficient water collection, thus providing technical feasibility for the engineering production and practical application of biomimetic spider silk materials.

Inspired by natural spiral structures, Wang et al. prepared helical-slot modified spindle-knot (HSK) microfibers through an innovative regular crack control strategy [88], leveraging the advantages of spiral structures for water collection (Figure 4i,j). In situ dynamic fog collection observations (Figure 4k) further verified the performance advantages of HSK. In a simulated low-humidity fog environment, the fog droplet capture rate of HSK is significantly higher than that of traditional non-grooved biomimetic fibers. Moreover, due to the water retaining effect of the spiral grooves, droplets on the fiber surface are less prone to evaporation in dry environments. Compared with smooth spindle-knot fibers, HSK fibers exhibit faster droplet coalescence and growth rates, higher water collection efficiency, and ultra strong water suspending capacity, demonstrating great potential for large-scale water collection applications.

To construct an effective internal structure for immediate water transport and enhance droplet collection efficiency, researchers have adopted electrospinning to fabricate interwoven fiber-structured materials. Liu et al. combined electrospinning with fluid coating to prepare bioinspired nanofibril-humped fibers (BNF) with strong water collection properties using thermoplastic polyester elastomer (TPEE) and chitosan (CS) (Figure 4l,m) [68,89,90,91,92]. This fiber features periodic humps composed of randomly interlaced nanofibers and nodes made of directionally aligned nanofibers, highly resembling the micro-nano structure of wet spider silk. Particularly, the interlaced nanofibers possess an extremely large specific surface area, which can effectively capture droplets and transport water through the channels between nanofibers under humid conditions, enabling BNF to rapidly capture droplets and immediately coalesce them into large droplets at the humps (Figure 4n). The capillary force generated at the junctions of the nanofiber protrusions and the nanofibers plays a crucial role in the wetting process, while the Laplace pressure difference dominates the internal transport process, significantly enhancing the fog collection efficiency. This provides inspiration for the design of new materials and can be applied in fog water collection engineering, filtration, and other fields.

#### 3.1.3. Synergistic Application of Biomimetic Spider Silk Fibers and MOF Materials for Atmospheric Water Harvesting

To expand the application scenarios of biomimetic water-collecting spider silk materials beyond fog collection, researchers have combined the directional transport advantage of biomimetic spider silk fibers with the efficient moisture absorption property of metal–organic framework (MOF) materials, developing novel composite water collection systems for atmospheric water harvesting. This integration significantly improves the water collection efficiency and practicality of the materials in dynamic environments [10,74,93,94,95,96,97,98].

**Figure 5:**
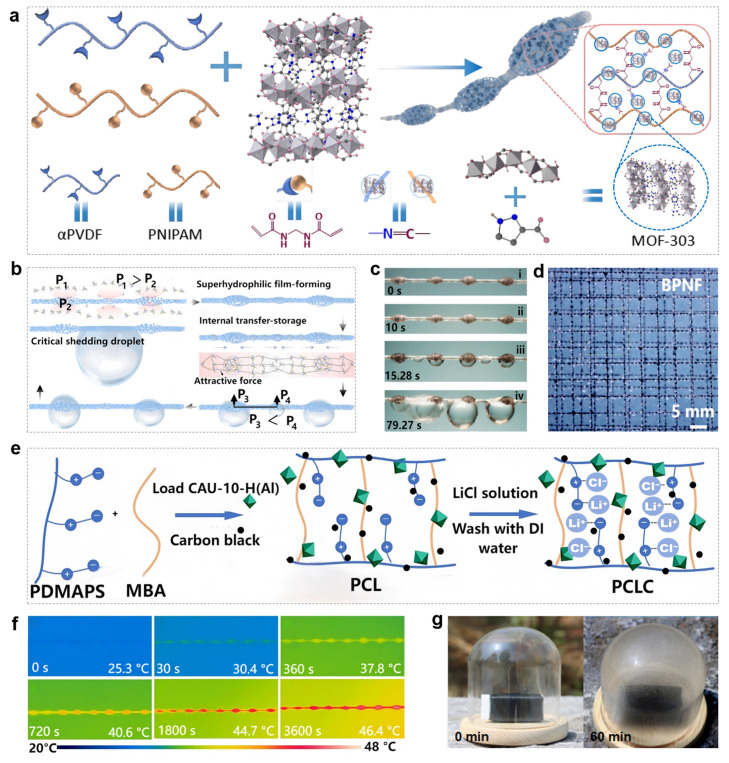
Synergistic application of biomimetic spider silk fibers and MOF materials for atmospheric water harvesting: (**a**) Schematic illustration of synthesizing BPNF; (**b**) Schematic representation of the atmospheric water collection mechanism of BPNFs; (**c**) Optical view of droplet capture and fusion on BPNFs; (**d**) Two-dimensional photo of suspended water droplets on BPNF following outdoor atmospheric water collection [74]; (**e**) Schematic diagram of the synthesis mechanism of zwitterionic hydrogel PCLC biomimetic fibers; (**f**) Infrared visualization of the temperature increase in a single PCLC fiber under a specific light intensity; (**g**) Optical image of water release from PCLC fibers when exposed to outdoor light [104].

Zhu et al. designed a bioinspired porous nanofibril-humped fibers (BPNF), composed of polyvinylidene fluoride (PVDF) grafted with poly(N-isopropylacrylamide) (PNIPAM) and in situ grown MOF-303 (Figure 5a) [74]. The water collection mechanism of BPNF is shown in Figure 5b. The surface pressure difference (P_1_ > P_2_) generated by the hump structure drives the directional adsorption of water molecules. And surface roughness and MOF-303 synergistically promote liquid film condensation. Stronger intermolecular forces in large droplets dominate internal water transport. The pressure difference between droplets triggers spontaneous merging, and the droplets continue to grow until reaching a critical volume, after which they fall due to gravity. The actual water collection process is shown in Figure 5c,d. By combining the spider silk-inspired hump geometric structure with advanced functional materials, BPNF achieves breakthrough performance in high-humidity atmospheric water harvesting. This integrated “adsorption–condensation–transport” design paradigm lays the fundamental principle and technical foundation for next-generation atmospheric water collection devices, and its morphological innovation opens a new path for enhancing water collection performance.

To further extend the application of biomimetic spider silk fibers to atmospheric water harvesting in arid regions with low humidity, Pan et al. introduced the zwitterionic hydrogel poly [2-(methacryloyloxy)ethyl]dimethyl(3-sulfopropyl) ammonium hydroxide (PDMAPS) and the MOF material CAU-10-H as carriers for the hygroscopic salt lithium chloride (LiCl), and added carbon black (CB) nanoparticles to finally prepare PDMAPS/CAU-10-H/LiCl/CB (PCLC) composite spider silk fibers (Figure 5e) [99,100,101,102,103,104]. The CB nanoparticles, acting as photothermal conversion agents, enable the conversion of light energy to thermal energy, thereby enhancing the water capture and release kinetics of PCLC spider silk. Figure 5f shows an infrared image of a single PCLC spider silk fiber under one sun irradiation. The continuous and significant temperature rise clearly demonstrates the fiber’s excellent photothermal effect. To systematically verify the operational efficiency of PCLC spider silk in practical atmospheric water collection, batch-fabricated PCLC spider silk fibers were placed in a water collection device for outdoor experiments, and the condensation process of water vapor on the fibers was observable through a glass cover (Figure 5g). Under sunlight irradiation, the water collection effect of the device was recorded over 60 min, and obvious droplets gradually formed on the container wall, confirming the material’s significant water desorption efficiency. The system can independently complete the “adsorption -irradiation release” cycle in outdoor dynamic environments, verifying the feasibility of outdoor water collection. This integration significantly improves the material’s environmental adaptability and energy utilization efficiency, providing a key technical path for the development of biomimetic water collection materials toward low energy consumption, high adaptability, and practicality.

### 3.2. Heterogeneous Wettability Surfaces Inspired by Desert Beetle Backs for Droplet Transport

**Figure 6:**
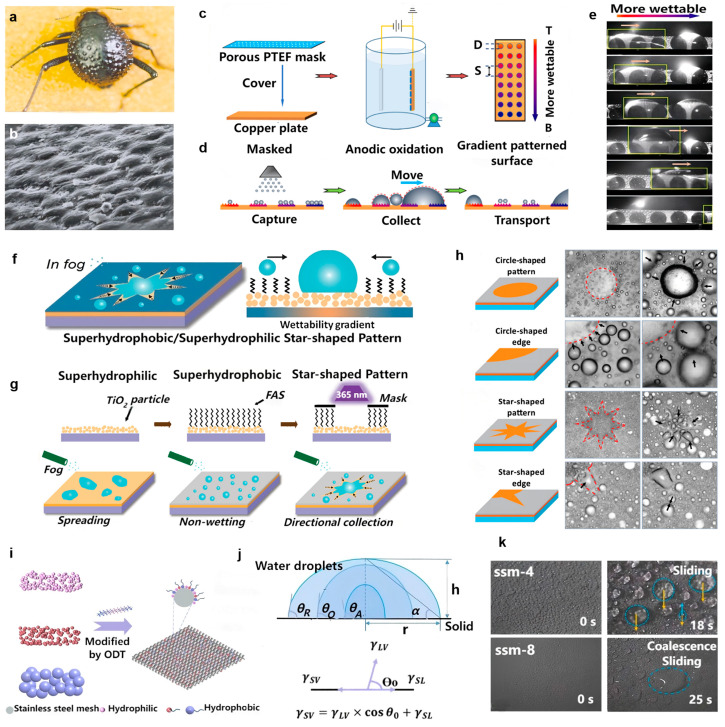
Heterogeneous wettable surfaces inspired by desert beetle backs for droplet transport: (**a**) Optical image of the desert beetle’s elytral surface; (**b**) SEM micrograph of the groove region, showing an array of flat hexagonal microstructures coated with wax [30]; (**c**) Schematic outline of the preparation procedure for the WPGS; (**d**) Schematic illustration of the three fog collection steps on the WPGS surface; (**e**) Schematic diagram of droplet movement on the WPGS surface [108]; (**f**,**g**) Schematic representations of the preparation process for the biomimetic surface with star-shaped wettability patterns; (**h**) Comparison of fog droplet collection behaviors on surfaces with varying wettability [105]; (**i**) Fabrication of stable superhydrophilic/superhydrophobic hybrid surfaces; (**j**) Wetting behavior of water droplets on a solid surface; (**k**) Optical photographs of the fog capture process on superhydrophilic/superhydrophobic hybrid surfaces ssm-4 and ssm-8 samples [111].

Desert beetles inhabit the Namib Desert on the west coast of Africa (Figure 6a). At night or during heavy morning fog, they climb to the tops of sand dunes and adopt a head-down posture to face the rising fog flow. When fog droplets accumulate on their backs, the droplets flow into their mouths along the back. The surface of the beetle’s back is irregularly convex-concave, consisting of alternately arranged hydrophobic waxy regions and hydrophilic non-waxy regions [44,45,105,106,107]. Magnified SEM images show that its microstructure comprises numerous hexagonal hemispheres (approximately 10 μm in diameter) (Figure 6b), forming a superhydrophobic system similar to that of lotus leaves. During fog water collection, fog droplets preferentially condense and grow on the hydrophilic protrusions of the desert beetle’s elytra, while the hydrophobic depressions guide the fog droplets toward the hydrophilic regions. When the droplets grow to a critical size that covers the entire hydrophilic protrusion, gravity overcomes capillary adhesion, causing the droplets to detach. The detached droplets roll down along the inclined elytra, and the hydrophilic protrusions not involved in condensation correct the trajectory with weak adhesion, ultimately guiding the droplets to converge.

Inspired by the synergistic water collection effect of different wettable regions on desert beetle backs, researchers have designed various materials for droplet transport and water collection in recent years. Drawing on the unique water collection capability (wettable patterns) of Namib desert beetles and wet-rebuilt cribellate spider silks (wettable gradient), Xing et al. developed an innovative biomimetic integrated surface with both wettable patterns and gradients (WPGS) through a novel one-step anodization process (Figure 6c) [108]. This surface not only enhances fog droplet capture performance via hydrophilic patterns but also maintains efficient water drainage through the hydrophobic substrate during fog collection. Figure 6d shows the three main steps of the fog collection process on the WPGS surface: capture, aggregation, and transport. First, droplets captured from the fog-laden airflow aggregate into larger droplets on the surface. Subsequently, the droplets are directionally transported to regions with higher wettability via the wetting gradient. This process accelerates surface regeneration, thereby improving water collection efficiency and enabling a continuous water collection cycle (Figure 6e).

Bai et al. designed a novel surface with star-shaped wettable patterns. A superhydrophilic surface was prepared by spin-coating titanium dioxide slurry on a bare glass substrate. The film was then treated with heptadecafluorodecyl-trimethoxysilane (FAS) to convert its wettability to superhydrophobic. Finally, selective ultraviolet irradiation with a photomask of a specific pattern was applied to obtain the desired wettable pattern characteristics (Figure 6f,g) [105,109,110]. By integrating the surface energy gradient and Laplace pressure gradient, this star-shaped wettable pattern surface can rapidly drive tiny water droplets toward regions with higher wettability, preventing them from being scattered by wind. Figure 6h shows the difference in fog droplet collection processes on surfaces with different wettabilities. On the surface with circular wettable patterns (marked by red dashed lines), the surface energy gradient drives tiny droplets in the superhydrophobic region toward the superhydrophilic region to form larger droplets. These droplets are easily aggregated from the outside and move directionally to the circular hydrophilic region, and a new collection cycle starts immediately after the droplets detach from the original region. On the surface with star-shaped patterns, the star tips generate a Laplace pressure gradient via the shape gradient, which further enhances the directional movement of droplets; the droplets move toward the center of the star-shaped pattern and spread, and a new collection cycle initiates immediately after the droplets leave their original positions. Within the same time period, the surface with star-shaped patterns exhibits higher water collection efficiency than that with circular patterns, providing important insights for the design and development of high-efficiency biomimetic water collection materials with controllable wettability.

To enhance nucleation efficiency during fog collection without compromising liquid transport performance, Wang et al. developed a novel spray-coating process [111]. By leveraging the synergistic effect of ZrO_2_ nanoparticles, micron-sized CuO particles, and nano-sized Cu_2_O particles, hybrid nano-superhydrophilic sites were constructed on a superhydrophobic surface (Figure 6i) [112,113,114]. By reducing the hydrophilic regions from the macroscale to the microscale, the resulting structured surface not only maintains excellent water-harvesting efficiency analogous to the elytra of beetles but also avoids the phenomenon of surface liquid film flooding. When droplets condense, the presence of nano-sized hydrophilic sites increases the proportion of the droplet’s contact area with the hydrophobic regions. Owing to the small contact area between the droplets and the hydrophobic material surface, the droplets form a more spherical morphology, which facilitates their sliding or rolling, shortens the initiation time of water droplets from capture to transport, reduces motion resistance, and thereby improves transport speed (Figure 6j). By adjusting the ratio of micro- and nano-particles to precisely regulate the morphology and wettability of the hybrid surface, the balanced optimization of surface nucleation and transport performance can be achieved (Figure 6k). This study not only provides a practical strategy for addressing global water scarcity but also offers a manufacturing technical solution for the industrial production of customized surfaces with micro-nano structures and non-uniform wetting patterns.

### 3.3. Biomimetic Materials Inspired by Cactus Spines for Fog Collection

**Figure 7:**
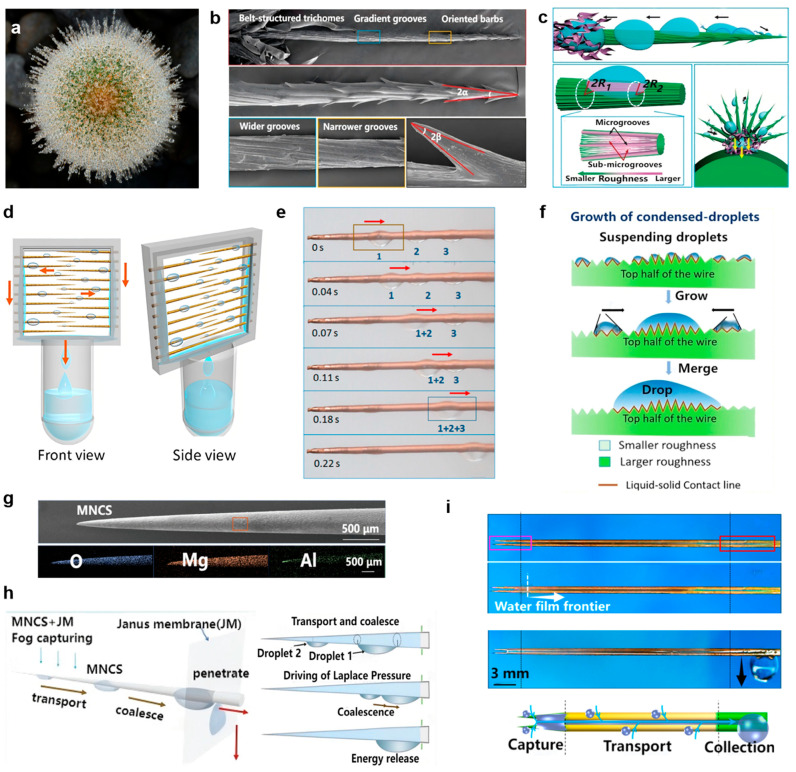
Biomimetic cactus spines for fog collection: (**a**) Optical image of cactus spines adorned with dew. Photo by Sebastian Schuster, Unsplash, https://unsplash.com/; (**b**) SEM micrograph of a single cactus spine; (**c**) Schematic illustration of the fog collection mechanism of cactus spines [34]; (**d**) Schematic diagram of the water collection device equipped with PCCWs array; (**e**) Process of droplet collection on PCCWs; (**f**) Schematic representation of the water collection mechanism of the PCCWs array [117]; (**g**) SEM image of the rough MNCS; (**h**) Schematic diagram of the droplet collection mechanism on MNCS [116]; (**i**) Process of liquid collection on the biomimetic integrated surface [119].

As a typical plant in arid and semi-arid regions, cactus spines have evolved through long-term adaptive evolution to achieve efficient fog droplet capture, directional transport, and aggregation, providing a biological prototype for addressing low water collection efficiency in low-humidity environments. In a foggy environment, a continuous and uniform dew layer forms on the surface of cactus spines, and droplets flow directionally from the tip to the base along the spine’s axial direction without obvious backflow or retention (Figure 7a). A single cactus spine consists of three regions: directional barbs at the top, numerous gradient grooves in the middle, and belt-shaped trichomes at the base (Figure 7b). The spiny conical main trunk has narrow parallel grooves at the top, which gradually transition to wider grooves at the base [23,29,34,40,115,116]. Figure 7c illustrates the fog collection mechanism of cactus spines. Droplets first deposit on the surface of the barbs and trichomes and move directionally along them. With continuous deposition and droplet merging, the enlarged water droplets detach from the spine tips. The larger droplets then continue to be transported along the gradient grooves, and are finally absorbed by the epidermal trichomes at the spine base. The Laplace pressure gradient generated by the conical structure of the trichomes and the surface free energy gradient generated by the roughness gradient along the trichome surface constitute the dual mechanical mechanisms driving the directional movement of droplets. Cactus spines integrate the functions of water deposition, collection, transport, and absorption, enabling an efficient fog collection process without relying on external energy.

Based on the structural characteristics of cactus spines, researchers have prepared a series of biomimetic water collection materials through multi-technology integration to achieve precise replication and performance optimization. Xu et al. proposed an artificial periodic roughness gradient conical copper wire (PCCW) fabricated via an electrochemical corrosion method controllable by a periodic current gradient. A schematic diagram of the PCCW array water collection device is shown in Figure 7d [117,118]. Under continuous fog flow impact, multiple droplets form on the high roughness regions (HRRs) of PCCW. When the droplets grow to the critical size for self-propelled movement, they start to move and merge with other droplets into larger droplets, which then quickly move toward the PCCW base (Figure 7e). This capability stems not only from the combined effect of the geometric curve gradient (generating Laplace pressure) and the periodic roughness gradient (generating surface energy difference) but also from the dynamic energy released by droplet deformation during coalescence (Figure 7f). This PCCW structure can capture fog droplets at periodic points on the conical surface from the air and realize long-distance droplet transport without external force, providing new ideas for future fog water collection projects.

Inspired by the integrated fog water collection system of cactus spiny trichomes, Zhou et al. proposed a design strategy for an integrated system (MNCS + JM) composed of micro/nanostructured conical spines (MNCS) and Janus membranes (JM) [116]. By constructing rough MNCS on the surface of conical spines, tiny fog droplets are captured, coalesced, and directionally transported (Figure 7g). When a Janus membrane (JM) with internal hydrophobicity and external hydrophilicity is vertically placed at different positions of MNCS, the water collection behavior during droplet transport can be precisely regulated, enabling the MNCS + JM system to promote droplets to undergo a continuous cyclic collection process of transport-coalescence-transport (Figure 7h). Owing to the synergistic effect of Laplace pressure difference and surface energy release during droplet coalescence, as well as the wetting force difference between the superhydrophobic and hydrophilic regions of the JM, higher-efficiency fog water collection is achieved, providing new insights for regulating droplet transport on material surfaces.

Through an innovative capillary-induced selective oxidation method, Wang et al. developed a novel integrated bioinspired surface (IBS). The conical capture region, featuring hydrophilicity and a multi-level structure, endows the surface with excellent fog droplet capture performance (Figure 7i) [119]. Benefiting from the wettable pattern gradient in the transport region and the superhydrophilic property in the collection region, captured droplets are confined in microchannels and rapidly transported to the collection region under the synergistic effect of capillary action and wetting gradient, thereby significantly improving the surface reconstruction rate. This integrated biomimetic surface design is expected to be applied in high-efficiency water collection systems, microfluidic devices, and other fields.

### 3.4. Rapid Fog Collection Inspired by Shorebird Beaks

**Figure 8:**
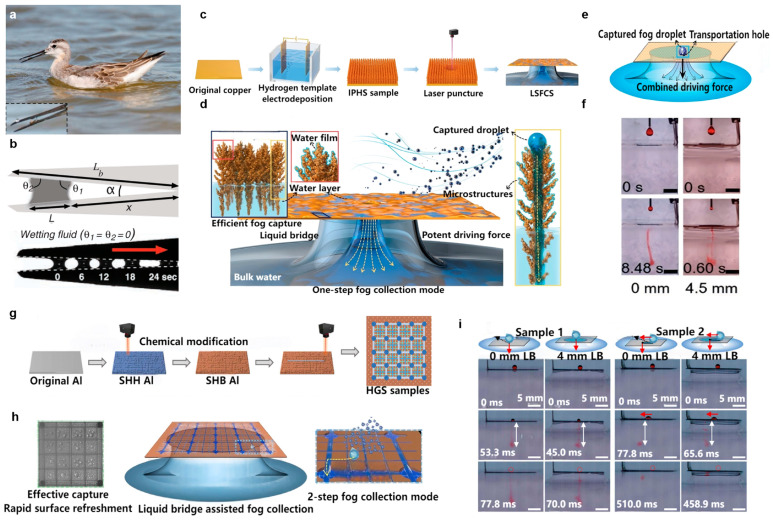
Biomimetic shorebird beak structures for rapid fog collection: (**a**) Optical photograph of a juvenile Wilson’s phalarope feeding (displaying the beak structure); (**b**) Schematic diagram of the shorebird beak and the movement of droplet transport between the upper and lower jaws [33]; (**c**) Schematic outline of the design and preparation of the LSFCS; (**d**) Schematic illustration of one-step fog collection using LSFCS; (**e**) Schematic representation of the one-step fog collection principle; (**f**) Optical images of droplet transport with liquid bridges of different lengths [120]; (**g**) Schematic diagram of the preparation procedure for the HGS samples; (**h**) Schematic illustration of the fog collection mode of the LBAFCS; (**i**) Droplet penetration on samples with different liquid bridge lengths [123].

In wetland ecosystems, shorebirds rely on the liquid bridge structure formed by the closure of their upper and lower jaws to overcome the dependence of traditional water collection materials on gravity. Through the synergy of liquid bridge tension and capillary force, they achieve efficient fog droplet convergence and transport, providing a biological prototype for the development of water collection materials in low-energy-consumption and dynamic fog environments. The shorebird beak has a wedge-shaped structure (Figure 8a), and efficient directional droplet transport is achieved through periodic opening and closing of the beak [33]. A fully wettable liquid is deposited in the wedge-shaped structure to form a droplet spanning the wedge-shaped region (Figure 8b). When the droplet propagates toward the narrow region, it advances at a constant speed initially and accelerates as it approaches the wedge apex. Emulating the liquid bridge structure of the beak to achieve efficient droplet transport, researchers have designed and fabricated a series of materials.

Chen et al. proposed a one-step fog water collection mode (Figure 8c) using liquid bridges in synergy with interconnected porous superhydrophilic structures (IPHS) fabricated via a hydrogen bubble template electrodeposition method—namely, the liquid bridge synergistic fog collection system (LSFCS) [120,121,122]. The liquid bridge provides a strong driving force during fog water collection, which significantly increases the liquid bridge-driven droplet transport speed. Benefiting from the superhydrophilic property of IPHS and the strong interaction effect of the liquid bridge, even if the lower part of IPHS is covered by a water layer during fog capture, its upper part can still protrude above the water surface to maintain a rough surface structure, thereby achieving stable and efficient fog capture performance (Figure 8d). The liquid film is connected to the water layer, liquid bridge, and bulk water, enabling the liquid bridge to exert force on the captured droplets through water connectivity. After being captured by IPHS, tiny fog droplets immediately merge with the thin liquid film and are synchronously transported to the underlying bulk water through the water layer, driven by the Laplace pressure difference between the droplets and the liquid bridge (Figure 8e). The resultant force acting on the liquid bridge in the z-direction (*F_z_*) is:

(8) $$F_{z}=\pi \;R_{\mathrm{Cu}}^{2}\;\Delta P_{1}+2\pi \;R_{\mathrm{Cu}}\;\gamma \;\sin\;\theta -\mathrm{mg}$$

where *R_Cu_* is the radius of the area of where the liquid bridge contacts the solid, Δ*P*_1_ is the Laplace pressure difference in the liquid bridge, *θ* is the contact angle of the liquid bridge on the sample, *m* is the mass of the liquid bridge, *g* is the acceleration due to gravity, respectively. Additionally, the driving force provided by the liquid bridge is positively correlated with its length, derived from the positive correlation between liquid bridge curvature and Laplace pressure. As the liquid bridge length increases, the transport speed increases accordingly (Figure 8f). This study provides new ideas for improving fog water collection efficiency and can be applied in condensed water collection or droplet manipulation fields.

Chen et al. also pioneered the combination of hierarchical grooved surfaces (HGSs) with liquid bridges (LBs) to design a liquid bridges assisted fog collection system (LBAFCS) [59,60,123]. A superhydrophilic (SHH) aluminum substrate was first prepared by laser etching a blank aluminum plate. The substrate was then converted to a superhydrophobic (SHB) aluminum substrate via chemical modification. Finally, secondary etching was performed to obtain a HGS sample (Figure 8g). The HGS sample exhibits both efficient fog capture capability and rapid surface renewal characteristics. The presence of the liquid bridge merges the coalescence and transport steps in traditional fog collection into a single removal step: when water droplets coalesce with the thin liquid film at the edge of the sample groove, they are synchronously transported to the container via the liquid bridge. This mechanism also converts intermittent droplet transport under gravity into a continuous process, eliminating the need to wait for droplets to grow to a specific volume (Figure 8h). The introduction of a 4 mm LB provides the sample with additional driving force derived from the Laplace pressure difference at its curved edge, thereby promoting droplet transport (Figure 8i). Rapid droplet transport increases the surface renewal rate, which in turn improves fog collection efficiency and enables efficient droplet collection. This study has potential applications in condensed water collection, microfluidics, and high-efficiency mass and heat transfer fields.

### 3.5. Applications of Water Collection Materials

Beyond classic biological prototypes, a diverse array of materials and mechanisms have been developed for enhanced water collection. These systems can be broadly categorized by their primary operating principles. One strategy leverages precise structural engineering to control liquid transport. Examples include nanocone arrays that capture and directionally transport tiny droplets towards the formation of a continuous water film, and three-dimensional fibrous networks that utilize Rayleigh instability and capillary forces between micro-wires for rapid liquid transport. Gradient surfaces, with spatially tuned wettability from hydrophobic to superhydrophilic regions, further enable controlled droplet motion. Another approach employs advanced material chemistry to optimize the adsorption and release of water vapor. This includes metal–organic framework (MOF)-based composites, such as MOF-303/thiolated chitosan (MTC), which achieve high-capacity water uptake at low relative humidity and efficient release at mild temperatures. Similarly, photothermal aerogels integrate biomimetic multi-channel architectures with solar-absorbing components, enabling cyclic water capture and solar-driven release. Together, these structural and chemical design paradigms significantly expand the toolkit for atmospheric water harvesting, offering tailored solutions for varying environmental conditions and application requirements.

**Figure 9:**
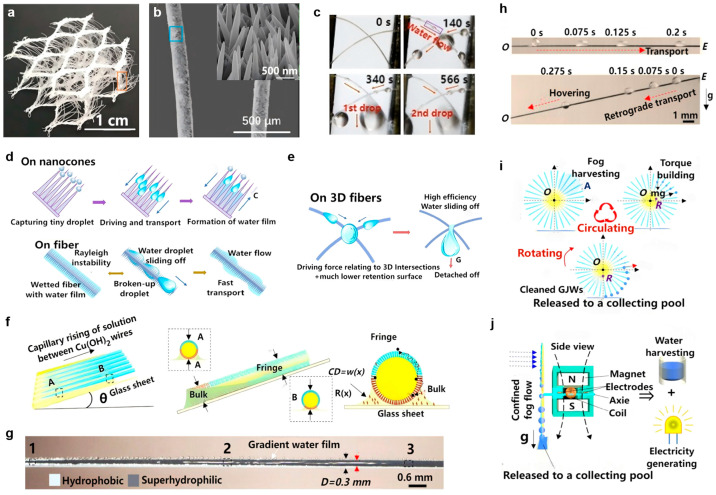
Materials for fog collection applications: (**a**) Optical photograph of the N3D; (**b**) SEM micrograph of a single fiber within N3D; (**c**) Optical view of the fog collection process of N3D; (**d**) Schematic illustration of the droplet transport mechanism on nanocone clusters and inclined fibers; (**e**) Schematic diagram of the droplet collection mechanism on 3D fibers [124]; (**f**) Optical microscopic view of Gradient-Janus wire (GJW) produced on a large scale; (**g**) Optical microscopic image of the fabricated gradient-junction silk; (**h**) Optical images documenting the transport and hovering of water droplets on the GJW surface over time; (**i**) Schematic representation of the operation of the fog-triggered rotating GJWs-wheel; (**j**) Schematic diagram of the fog-triggered GJWs-wheel power generation device [125].

Li et al. achieved biomimetic design by adopting a three-dimensional multi- intersection network structure and modifying it with hydrophilic zinc oxide nanocones (i.e., N3D) (Figure 9a,b) [75,117,124]. For the intersecting fibers of N3D, the hydrophilic zinc oxide nanocones capture water molecules, rapidly forming tiny water droplets that tend to flow directly to the intersection points and slide to the junctions. Meanwhile, the growing water droplets slide and merge, forming larger droplets at the intersections that fall off the fibers, accelerating the fog collection rate (Figure 9c). This multi-intersection network can effectively guide directional water transport: when the nanocones cooperate with the three-dimensional fiber network, the tangential retention force is weakened by the combined effect of Laplace force, droplet weight gain, and Rayleigh instability, allowing trace water to form a continuous water flow rather than remaining on the N3D surface (Figure 9d,e). This material not only enables larger-scale water collection but also its nanocone decoration layer significantly improves water collection efficiency, creating a new fog collection mode that synergistically enhances fog droplet capture and water transport. It can provide practical references for research in fields such as microfluidics, catalysts, and batteries. The N3D material can be applied in production and daily scenarios such as cooling tower steam recovery, agricultural irrigation systems, and water-scarce countries to realize the practical application of large-scale fog water collection.

To design a fog water collection system that balances energy development and water resource acquisition, Zhong et al. prepared Gradient-Janus wire (GJW) using a liquid-confined modification method (Figure 9f) [125,126,127]. This material has opposite conical wetting characteristics arranged along the same direction—i.e., the superhydrophilic region increases while the hydrophobic region decreases (Figure 9g). This design enables GJW to achieve more versatile droplet manipulation functions: on one hand, Laplace force promotes directional droplet transport along the gradient; on the other hand, this force hinders reverse droplet transport. This feature allows GJW to exhibit controllable water suspension, transport, and pinning phenomena during fog water collection (Figure 9h). A wheel composed of multiple radially arranged GJW can be designed as an efficient rotating structure, with its driving force derived from the torque generated by fog water collection (Figure 9i). The slender GJW with a small diameter has a lightweight structure, enabling it to operate with a minimal amount of fog water. With continuous collection, droplets form on the GJW surface, and each droplet generates torque to drive the wheel rotation. When the total torque overcomes resistance, the GJWs-wheel starts to rotate. Benefiting from the superhydrophilic/hydrophobic conical gradient region on the same side of GJW, all collected droplets can be precisely manipulated to anchor at a certain point on the rotating GJW and released into the water collection tank when rotated to a specific position. This continuous collection-release cycle mechanism enables simultaneous power generation and water collection (Figure 9j), providing new ideas for the design of fog-triggered functional materials.

**Figure 10:**
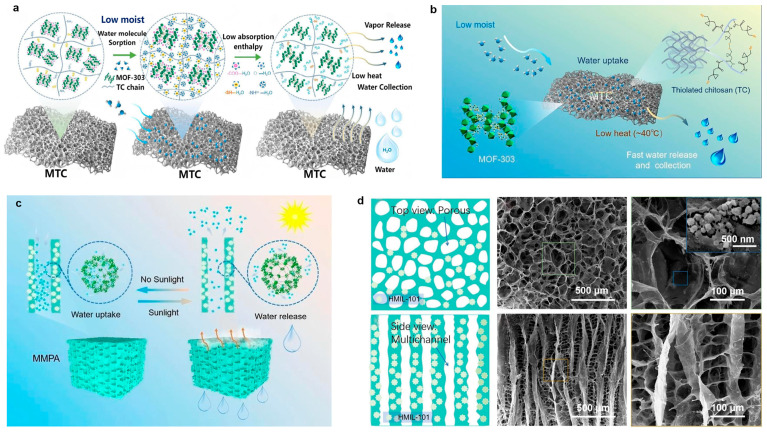
MOF-based composite materials for water collection: (**a**) Schematic of the water harvesting process in MTC; (**b**) Schematic Diagram of MTC Composition [97]; (**c**) Design Schematic of the MMPA; (**d**) Illustration and SEM images of MMPA in top view and side view [128].

To achieve long-cycle life and high-capacity water harvesting under low relative humidity conditions, Luo et al. developed a composite material (MTC) consisting of MOF-303 and thiolated chitosan (TC) [97]. By regulating the reaction conditions between the thiol groups on the polymer chains and the MOF-303 crystals, MOF-303 was uniformly distributed within the TC framework, forming a porous structure that significantly enhances moisture capture capability in low-humidity environments (Figure 10a,b). The study emphasizes that a multivariate strategy-optimizing thiol group content and preparation methods-effectively tunes the material’s hydrophilicity and water absorption performance. MTC, prepared via thiol modification and blend doping, features a porous architecture that broadens its water absorption range and reduces operational limitations. The van der Waals forces and hydrogen bonding between TC and MOF-303 reduce the energy required for water desorption, enabling a water release capacity of 0.36 g/g at approximately 40 °C. This research provides new insights for developing intelligent desiccants and water engineering systems for efficient atmospheric water harvesting.

Inspired by the multi-channel structure of conifer xylem, Gao et al. developed a photothermal aerogel (MMPA) using hollow MIL-101(Cr) as the substrate, fabricated via directional freeze-drying and Ca^2+^ ion cross-linking techniques [128]. The material integrates hollow MIL-101(Cr) (HMIL), polypyrrole-modified cellulose nanofibrils (CANFs), and sodium alginate (SA) to form a hierarchical micro-nanochannel network with aligned micropores (Figure 10c). The HMIL particles, uniformly distributed within the channel layers, endow the aerogel with rapid moisture adsorption and efficient solar-driven desorption capabilities. Also, the MMPA exhibits a hierarchical multi-level pore channel structure (Figure 10d). Its micropores, measuring approximately 100 to 300 μm in size, are enveloped by a continuous layered architecture, presenting a morphology analogous to a honeycomb-like porous network. This porous structure is interwoven with sheet-like layers, and HMIL particles are uniformly distributed on the surfaces of these constituent channel-forming layers. Side-view SEM images further reveal an anisotropic multi-channel architecture. These channels are separated by lamellar gaps with thicknesses ranging from about 100 to 500 μm, forming a unique array-like channel system where the sheet-like structures are observable acting as bridges between channels, collectively constructing an integrated porous structure. This design highlights the potential of scalable, practical materials for high-efficiency atmospheric water harvesting applications.

Biomimetic water-harvesting materials exhibit substantial application value across diverse practical scenarios: Wu et al. developed slippery liquid-infused porous surfaces (SLIPS) inspired by biological prototypes such as desert beetles and cactus spines, preserving hydrophilic bumps via an underwater oil infusion strategy [129]. These surfaces achieve efficient water harvesting at fog flow rates of 300–1500 mL/h, and integration with TiO_2_ photocatalysts enables synchronous pollutant degradation, rendering them suitable for water purification in atmospherically polluted regions. Zhou et al. fabricated bioinspired slippery harp structures (HSFC) mimicking the surface properties of Nepenthes pitcher plants and desert beetles [130], modified with silica gel, the water collection rate is significantly enhanced by 60% compared to hydrophilic surfaces, finding utility in atmospheric water generation, industrial steam recovery, and related fields. Additionally, Zhou et al. designed a bioinspired dual-function device (BDFD) integrating fog harvesting and hydro-to-electricity conversion [131], with a water collection efficiency 305% higher than that of non-biomimetic samples and a stable output voltage of 12.5 V, offering a promising solution for sustainable water and energy supply in more regions worldwide. In agricultural contexts, Zhu et al. utilized condensed water collected by bioinspired porous nanofibril-humped (BPNF) three-dimensional network structures for precise drip irrigation and crop cultivation [74]. The collected water quality effectively supports high-efficiency crop growth, and the system consumes lower energy compared to traditional irrigation methods, providing a sustainable approach to address water scarcity in arid regions.

The research progress on the application of biomimetic water collection materials is summarized in Table 1.

## 4. Summary and Future Direction

In summary, natural organisms have evolved exquisite structure–function synergies for water harvesting through long-term adaptive evolution, providing unparalleled biological templates and mechanistic insights for the development of biomimetic water-collecting materials. Spider silk’s periodic spindle-knot–joint architecture, the heterogeneous wettability pattern on desert beetle elytra, the gradient grooved structure of cactus spines, and the liquid bridge-assisted transport mechanism of shorebird beaks collectively constitute the core design paradigms for biomimetic systems. By decoding and replicating these natural principles, researchers have advanced a diverse portfolio of biomimetic materials—ranging from fiber-based systems and heterogeneous wettability surfaces to integrated multifunctional devices and MOF composites—that exhibit remarkable performance in fog capture, directional droplet transport, and atmospheric water harvesting. These materials have demonstrated substantial potential in practical scenarios, including arid region water supply, agricultural irrigation, industrial water recovery, and portable emergency devices, laying a solid foundation for addressing global water scarcity.

Notably, the field has progressed from simple replication of single biological structures to the integration of multiple natural principles, enabling synergistic optimization of capture efficiency, transport speed, and environmental adaptability. For instance, the combination of spider silk-inspired gradient structures with MOF materials has expanded applicability to low-humidity environments, while liquid bridge-assisted systems have overcome the reliance on gravity-driven transport, unlocking new possibilities for low-energy-consumption water collection. Such advancements underscore the value of bioinspiration in driving material innovation, bridging the gap between fundamental interfacial science and engineering applications.

Despite these achievements, significant challenges remain for the translational deployment of biomimetic water-collecting materials. First, most high-performance materials rely on precision fabrication techniques and complex processes, resulting in high production costs and difficulties in ensuring structural consistency during large-scale manufacturing. Second, environmental stability remains a critical bottleneck—exposure to harsh conditions such as high dust, extreme temperature fluctuations, and strong winds often causes structural deformation, wettability gradient degradation, or functional failure. Third, the integration of water collection, transport, storage, and utilization into a complete system is insufficient, limiting adaptability to real-world application demands.

Future research should focus on targeted innovations to address these barriers. Priority should be given to developing low-cost, large-scale green fabrication technologies—such as roll-to-roll processing, parallel-nozzle microfluidics, and scalable electrospinning—to balance performance stability with cost reduction. Enhancing environmental adaptability requires the integration of functional components: self-cleaning coatings (e.g., titanium dioxide nanolayers) to mitigate dust accumulation, shape memory polymers to counteract structural deformation, and photothermal conversion materials to improve low-humidity performance. Furthermore, in-depth customization for specific scenarios is imperative—tailoring structural designs and system integration for arid regions (low-humidity adsorption–desorption cycles), offshore platforms (anti-salt fog corrosion), and outdoor emergencies (miniaturized integrated devices) will accelerate the translation from laboratory research to engineering practice. Interdisciplinary integration will also drive breakthroughs. Combining material science with ecology to decipher unexploited biological water-collection mechanisms (e.g., from lesser-studied organisms in extreme environments) can inspire novel structural designs. Merging nanotechnology with energy engineering will advance multifunctional devices, such as systems integrating water harvesting with electricity generation or solar-driven desalination. Collaborations with agricultural and civil engineering will facilitate the integration of biomimetic materials into irrigation systems, building facades, and urban green infrastructure, expanding application boundaries.

## Figures and Tables

**Table 1:** The application of biomimetic water collection materials.

Biomimetic Water Collection Materials	Bioinspired Organisms	Performance	Ref.
HDSSF	Spider silk and Sarracenia trichome	Water collection rate 9.03 g cm^−2^ h^−1^	[78]
HMSSF	Spider silk and Sarracenia trichome	Fog collection rate 0.34 μL s^−^^1^	[82]
HRSF	Spider silk	Water collection velocity 1.47 g h^−1^ (fog flow rate 3.9 mL min^−1^)	[83]
HSK	Spider silk and helical structure	Fog collection rate 0.22 μL s^−1^	[88]
BNF	Spider silk	Water collection efficiency 1.083 μL s^−1^ mm^−2^ (fog flow rate 150 g s^−1^ m^−2^)	[68]
BPNF	Spider silk	Water collection rate 1.63 g cm^−2^ h^−1^ (90–100% RH)	[74]
PCLC	Spider silk	Moisture absorbency 1.49–1.99 g g^−1^ (40% RH)	[104]
WPGS	Desert beetle back and spider silk	Water collection efficiency 0.2568 g cm^−2^ h^−1^	[108]
FAS	Desert beetle back	Water collection efficiency 2.11–2.78 g cm^−2^ h^−1^	[105]
Hyperphilic/Hydrophobic hybridized surfaces	Desert beetle back	Water collection efficiency 554.24 mg cm^−2^ min^−1^	[111]
PCCW	Cactus spine	Water collection efficiency 0.618 g cm^−2^ h^−1^ (fog velocity 2.4 m/s, 90%RH)	[117]
MNCS	Cactus spine	Water collection velocity 4.67 g h^−1^ (95% RH)	[116]
IBS	Desert beetle back, spider silk, cactus spine and Sarracenia trichome	Transport velocity 34.10 mm s^−1^	[119]
LSFCS	Shorebird beak	Fog collection efficiency 6.5 kg m^−2^ h^−1^	[120]
LBAFCS	Shorebird beak, desert beetle back and Sarracenia trichome	Fog collection efficiency 14.7 kg m^−2^ h^−1^	[123]
N3D	Spider silk	Fog collection efficiency 865.1 kg m^−2^ day^−1^	[124]
GJW	Spider silk and cactus spine	Output peak power 0.25 μW Water collection efficiency 38 ± 2.12 mg min^−1^	[125]
MTC	—	Moisture absorbency 0.135 g g^−1^ (12.5% RH)\Water release capacity 0.36 g g^−1^ (40 °C)	[97]
MMPA	Conifer xylem	Water uptake 7.84 L kg^−1^ day^−1^ Water harvesting ability 5.49 L kg^−1^ day^−1^ Average water release rate 0.37 L m^−2^ day^−1^	[128]
SLIPS	Nepenthes pitcher plant and desert beetle back	Water collection efficiency 5000–60,000 mg cm^−2^ h^−1^ (fog flow rate 300–1500 mL h^−1^)	[129]
HSFC	Nepenthes pitcher plant and desert beetle back	Water collection efficiency 25.1 mg cm^−2^ min^−1^	[130]
BDFD	Cactus spine and Sarracenia trichome	Water collection efficiency 48,940 mg cm^−2^ h^−1^ Transfer charge 28.9 nC	[131]

## Data Availability

Data will be made available on request.
